# *Autism Voices*: A novel method to access first-person perspective of autistic youth

**DOI:** 10.1177/13623613211042128

**Published:** 2021-09-04

**Authors:** Valérie Courchesne, Rackeb Tesfaye, Pat Mirenda, David Nicholas, Wendy Mitchell, Ilina Singh, Lonnie Zwaigenbaum, Mayada Elsabbagh

**Affiliations:** 1 McGill University, Canada; 2 The University of British Columbia, Canada; 3 University of Calgary, Canada; 4 University of Oxford, UK; 5 University of Alberta, Canada

**Keywords:** adolescents, autism, first-person perspective, lived experience, qualitative research, semi-structured interview, strength-oriented method, universal design

## Abstract

Opportunities to communicate first-person perspectives are essential for self-determination. However, many autistic youth are excluded from sharing their perspectives, specifically those who are minimally verbal or with lower intellectual functioning. Current challenges to capturing their voices include a lack of appropriate inclusive methodologies. Propose an inclusive strength-oriented method to capture first-person perspectives of autistic adolescents. Our protocol (“Autism Voices”) includes a pre-interview survey and semi-structured interview using universal design strategies. It was piloted with 33 participants who were representative of diverse language and cognitive abilities. A coding scheme was developed to identify communicative acts used by participants and mitigation strategies used by interviewers to enhance communication. Interviewer strategies that enhanced communication included question formulation, use of pictures, offering various output modalities, and flexible implementation of the protocol. Non-verbal and alternative communication responses (e.g. choosing to not respond) were informative to youth’s lived experience, especially for minimally verbal participants. Overall, our results highlight that communication goes beyond verbally answering questions and that participants’ unconventional communication conveyed rich information. *Autism Voices* provides a promising method to promote the inclusion of autistic youth in research.

**Lay abstract:**

The perspective of autistic individuals is often left uncaptured, and as a result they are often excluded from making decisions that impact them. Conventional communication can be challenging for many autistic individuals, especially those who are minimally verbal or who have an associated intellectual disability. Currently, a lack of appropriate methods to capture voices across the spectrum is a barrier. In the present study, we developed the *Autism Voices* protocol using universal design principles to capture the perspectives and experiences of autistic youth with a range of language or intellectual abilities. This protocol was then used with 33 autistic youth aged 11 to 18 years. A scoring rubric was developed to capture the unconventional communication used by the participants and the mitigation strategies used by interviewers to facilitate the interview. Many components of the protocol were found to effectively facilitate communication between the participant and interviewer, including the use of picture cards to support verbal questions/prompts, the fact that participants could respond with their preferred communication methods (writing, texting, pointing), and the fact that interviews were applied flexibly to adapt to each participant. Unconventional communication and mitigation strategies were mostly observed in interviews with minimally verbal individuals, but a fine-grained analysis showed participants were still communicating something through this unconventional communication. Our protocol could help promote the inclusion of more autistic individuals in research and showed that unconventional modes of communication like echolalia provide an understanding that participants’ are invested in conversations and certain topics are more meaningful than others.

## Background

For over two decades, the importance of involving children with disabilities or special needs in every stage of research has been recognized (Minkes et al., 1995; Rodgers, 1999; Stalker, 1998). The relevance of research, quality of the data collected, and application of findings into real-world settings are positively impacted when disabled people are closely involved in the research process (Carrington et al., 2016; Grinker et al., 2012; Inglis & Cook, 2011; Parr, 2016; Parsons & Cobb, 2013; Stalker, 1998). Moreover, their involvement has a positive impact on their own feelings of self-worth (Inglis & Cook, 2011). All children, including those with disabilities, have a right to self-determination (United Nations Children’s Fund, 1989). Hence, actively engaging youth with disabilities in research concerning them is their right (Stalker, 1998). However, researchers have historically interpreted the challenges encountered in participatory research with disabled youth as constraints related to their underlying diagnoses, instead of reflecting limitations inherent to previous methodological approaches (Booth & Booth, 1996).

Autism spectrum disorder (ASD), as described in the *Diagnostic and Statistical Manual of Mental Disorders* (5th ed.; DSM-5; American Psychiatric Association, 2013), includes individuals with various levels of adaptive functioning, language, intelligence, and symptom severity. This heterogeneity within the autism spectrum leads to uneven opportunities both in life and in research (Graetz, 2010; Jack & Pelphrey, 2017). To be able to alleviate the barriers to self-determination faced by many autistic individuals, we have to consider the personal limitations that may arise from autism or co-occurring conditions, in addition to contextual limitations that may be linked to non-optimal environments regardless of one’s diagnostic label(s) (Bronfenbrenner, 1989; World Health Organization (WHO), 2002). This is particularly relevant in autism, as diagnostic labels do not predict functional outcomes (WHO, 2002).

Despite the growing research in the autism field, meaningful participatory research has been lacking (Fletcher-Watson et al., 2018) and many decisions about how to support autistic individuals are determined without their active participation. Similarly, research on lived experiences in autism has predominantly focused on the accounts of parents, siblings, teachers, and clinicians, but unfortunately has not substantially included the voices of autistics themselves (Nicholas et al., 2019; Richards & Crane, 2020; Tesfaye et al., 2019). For example, a meta-analysis identified only 33 studies published between 1980 and 2014 focusing on the lived experience of autistic individuals, most of which involved verbal autistic adults with no intellectual disability (DePape & Lindsay, 2016). The participation of autistic people across the entire spectrum in research is important to orient authentic research questions and objectives; that is, those with lived experience who ultimately have the most at stake, rather than by researchers and funders (Fletcher-Watson et al., 2018). Representation across the spectrum is needed to promote inclusive research protocols, while also helping to guide policies that impact autistics (Perry-Hazan, 2016). This, in turn, can empower autistic individuals to contribute more widely to their environment and society, and to instill a strong sense of belonging (Andersen & Dolva, 2015).

A scoping review that we previously published highlighted that eliciting first person perspectives of people with autism in particular and those with disability in general bears many challenges (Tesfaye et al., 2019). Perspectives of minimally verbal autistics or autistics with lower IQ scores are largely uncaptured (Nicholas et al., 2019). In fact, these subgroups are understudied in general (Jack & Pelphrey, 2017; Kasari et al., 2013; Russell et al., 2019). However, the inclusion of other disabled children who share similar language or behavioral challenges has been done successfully (Bailey et al., 2015; Tesfaye et al., 2019). By using multiple facilitation strategies, such as visual supports, photographs taken by participants, drawing, as well as prompts and reformulation of questions, researchers have been successful in engaging disabled or pre-verbal children in research (Booth & Booth, 1996; Inglis & Cook, 2011; Minkes et al., 1995; Nind, 2008; Richards & Crane, 2020; Rodgers, 1999; Stalker, 1998). Successful research conducted with children with various disabilities, as well as research on autistic cognition and advocacy from autistic individuals, has started to shift the mistaken assumption that those who are minimally verbal or intellectually disabled are not able to offer valid perspectives (Chown et al., 2017; Lebenhagen, 2019; Rodgers, 1999).

In the current study, we build on these promising methodological developments by identifying an array of strategies that can potentially prove effective in capturing first-person perspectives of autistic teenagers. We developed and tested a novel approach called *Autism Voices*. We focused on capturing various aspects of their lived experience, including barriers and facilitators to participating in daily life in the way that they would like.

## Objectives

The objective of *Autism Voices* was to develop a method to capture first-person perspectives of autistic adolescents. We further aimed to document the efficacy of *Autism Voices* for youth participants with various levels of language and functioning abilities.

## Methods

### Participants

Participants were initially drawn from the Montreal and Edmonton cohorts of the *Pathways to better outcome in ASD study* (hereafter, “*Pathways*”), a multisite longitudinal Canadian study. *Pathways i*s an inception cohort study, thus creating a heterogeneous sample representative of the entire autism spectrum. *Autism Voices* was approved by the Research and Ethics Boards at McGill University and at the University of Alberta as an addendum to the *Pathways* study. Informed consent/assent was obtained from parents/guardians and participants. See the procedure section below for more details.

Participants of the *Pathways* study were enrolled at age of diagnosis (between 2:0 and 4:11 years old), and met the criteria for ASD on the Autism Diagnostic Observation Schedule (ADOS: Lord et al., 2000), in at least the social and one other domain of the Autism Diagnostic Interview-Revised (ADI-R: Lord et al., 1994), and based on *Diagnostic and Statistical Manual of Mental Disorders* (4th ed.; DSM-IV criteria; American Psychiatric Association, 2000), as recruitment was completed prior to the introduction of DSM-5. Children with known genetic, chromosomal, or neuromotor disorders were excluded, as were those with hearing or vision impairment. For the present study, active participants in the *Pathways* study and some participants who had declined participation in previous timepoints, but gave permission to be contacted for future studies, were contacted to participate. No other exclusion criteria were applied.

Of the 53 potential participants contacted, 62% (*n* = 33) agreed to participate. This included one of eight families who had declined participation at previous time points of the *Pathways* study. Thirty-three interviews were completed; however, one interview was excluded due to video capture failure. The final sample included 32 adolescents (6F: 26M), ranging from 11 to 18 years of age and with a broad range of verbal and intellectual abilities. Participant characteristics are presented in Table 1.

**Table 1.** Participant characteristics.

Measure	Mean (*SD*)	Range
Age (in years)	15.41 (2.26)	11–18
NVIQ	96.94 (22.30)	33–152
ADOS Social Communication	8.57 (3.74)	3–16
ADOS Restricted/Repetitive Behaviors	4.17 (3.71)	0–10

Note. NVIQ is expressed as a standard score and was estimated using the Perceptual Reasoning index of the Wechsler Abbreviated Intelligence Scales or Weschler Children Intelligence Scales, except for one participant for whom NVIQ was assessed with Leiter-R. Social Communication and Restricted/Repetitive Behaviors are algorithm scores are from the most recent administration of the Autism Diagnostic Observation Schedule (Lord et al., 2000). NVIQ: Nonverbal Intelligence Quotient; ADOS: Autism Diagnostic Observation Schedule.

The ADOS has different modules that can be administered that are dependent on a participant’s developmental and language level. Module 1 is intended for participants who do not use phrase speech (*n* = 2), Module 2 for those who use phrase speech but are not verbally fluent (n = 4), and Modules 3 and 4 are for those who are verbally fluent. Module 3 is best suited to children and youth <16 years of age (*n* = 14), and Module 4 is most appropriate for older adolescents and adults (*n* = 10). Based on the ADOS module administered and the NVIQ score, participants were sorted into one of four categories: low nonverbal intelligence and minimally verbal, low nonverbal intelligence (i.e. NVIQ < 85) and verbal, average/high nonverbal intelligence and minimally verbal, and average/high nonverbal intelligence and verbal. Participants who were administered ADOS modules 1 and 2 were considered minimally verbal and those who completed modules 3 and 4 were considered verbal. NVIQ was considered Average/High if they scored 85 or above. Two participants did not complete a recent ADOS as they opted out of the time-points when it was administered; hence, their scores are not reported. One of these participants spoke fluently and had been administered Module 3 at age 12, while the other did not and was administered Module 1 at the same age; therefore, they were considered to be verbal and minimally verbal, respectively. For all other participants, the ADOS was administered either on the same day (*n* = 7), or between 1 and 13 months of the study (n = 23). Given the stability of ADOS scores (Bieleninik et al., 2017), we considered the most recently obtained scores to be representative of the current level of symptoms (Table 1). Table 2 presents the number of participants in each of the aforementioned category.

**Table 2.** Number of participants per group of verbal and intellectual abilities.

	Low NVIQ	Average/High NVIQ
Minimally verbal	*n* = 4	*n* = 3
Verbal	*n* = 4	*n* = 21

NVIQ: Nonverbal Intelligence Quotient.

### Interview

We first identified themes to explore with the youth based on the self-determination (Deci & Ryan, 2004), ecological theory (Bronfenbrenner, 1989) and International Classification of Functioning (WHO, 2002) frameworks, as well as previous studies capturing the lived experience of children with neurodevelopmental disorders (DePape & Lindsay, 2016; Singh, 2011; Tesfaye et al., 2019). This led to the selection of six general themes: Community, School, Family, Future, Autism Experience, and Service Utilization. Questions were then developed around these themes. For example, a question asking participants to define their “super powers” was included to explore perceived self-competency, which is important to both self-determination and empowerment (Charlton, 2000). The need for connection, a core component of self-determination (Deci & Ryan, 2004), was touched upon through the exploration of various ecological systems within the participants’ life (family, friends, school, and community) (Gal, 2017). Moreover, the very acts of establishing rapport with the participants and conveying that their input and experiences were valuable, is in line with the goal of promoting self-determination. The resulting list of themes and questions was then reviewed in collaboration with other members of the research team, including expert clinicians and researchers in autism.

As a result of these incremental steps, a number of modifications were made. First, the *Service Utilization* theme, which aimed to understand participants’ experiences with health care services, was dropped because, as adolescent, they had less autonomous experiences with these services. The *Community* theme was re-labeled *Leisure*, to include information about participants’ interests and what they enjoyed (e.g. recreational activities). This change also allowed for teenagers who were less involved in their community to discuss their interests and leisure activities regardless of whether these were pursued within the community or at home. In addition, questions about emotions that encompassed multiple themes were added, along with reflective *wrap up* questions (e.g. what youth would change in their life, what would be involved in their “best day” ever, and what they thought was their “superpower”).

#### Parent survey

Interviewers worked in partnership with participants’ caregivers/parents to tailor the interview to each child’s profile and preferences. To develop a first version of the parent survey, information was also integrated from a prior consultation phase (via focus groups) with parents who identified possible barriers to their child’s participation as well as anticipated mitigation strategies (Tesfaye et al., 2019). Caregivers were surveyed about their child’s preferred methods of communication, living situation, interests, triggers (i.e. topics and interview approaches that might be upsetting), and preferences prior to each interview (see supplementary material for the full survey). In addition to informing how to tailor the interviews, the parent survey provided context from which the interviewer could confirm participants’ understanding and responses. For example, if the interviewer knew the participant did not have a sister, but the participant selected “sister” in response to the question “who is in your family?,” the interviewer could consider asking the question a different way and/or probe what was meant by the response of “sister.” This allowed for crosschecking of information and helped the interviewer to be more prepared to meet the participant, which are important components needed to conduct interviews with disabled youth (Rodgers, 1999; Stalker, 1998).

#### Pilot

A first draft of the interview protocol, including the pre-interview parent survey, was piloted with a minimally verbal participant. His mother provided feedback to the team behind a one-way mirror. Reported satisfaction and level of engagement demonstrated by the child and his mother suggested that the interview length (around 60 min) was adequate and could be attempted with other minimally verbal autistic teenagers. Minor edits were made to the pre-survey parent interview and semi-structured interview protocol. This included broadening the range of possible response outputs, such as responses that could be clarified by sorting cards (e.g. least to most) and cards for selecting binary options (yes/no and I like/I don’t like). These final edits led to the *Autism Voices* interview that was used for the present study.

#### Communication modalities

In order to make the interview as inclusive as possible, questions were asked using various modalities according to participants’ preferred method of communication, which were identified in the aforementioned parent survey. The modalities used by participants to answer questions also varied and included an augmentative and alternative communication (AAC) device, writing, drawing, texting, using emojis, choosing or pointing at pictures that were provided by the interviewer, and speaking. These modalities have previously been used successfully to interview youth with complex communication needs (e.g. Minkes et al., 1995; Nind, 2008; Rodgers, 1999; for a synthesis see: Tesfaye et al., 2019). Furthermore, picture cards were used with both verbal and minimally verbal participants. For example, some participants were provided with picture cards that they could sort by order of preference or into categorical piles such as “things I like to do” and ‘things I don’t like to do.’ Picture cards were used with verbal participants to support questioning by the interviewer and to suggest response options for participants who were unable to generate answers without prompts. Picture card prompts represented large categories (e.g. “health care”) to help participants better understand what types of response could be given. Furthermore, specific examples of answers (e.g. “nurse”) could also be provided when other prompts were insufficient. While this could introduce response bias, participants had to choose between options; therefore, there was still an element of agency in their answers. Such variations allowed us to tailor the interviews to individual preferences and abilities.

#### Procedure

Informed consent is essential when conducting research with disabled youth. Research Ethics Board committees often require researchers to obtain consent from a legal guardian or representative, before trying to obtain it from the youth themselves (Rodgers, 1999). Researchers have challenged this practice and suggest consent from youth should always be obtained first if possible (Rodgers, 1999; Stalker, 1998). In the present study, the Research ethic board also required that consent be obtained from a parent or legal guardian first as all participants (except one) were minors and as parents themselves were participants in our study. Hence, consent was obtained from parents of minors and assent was then obtained from youth participants. The only exception was for the participant who was 18 years old, who was able to consent for himself. For the assent process, as suggested by Rodgers (1999) and inspired by Harrington et al. (2014), various adaptations to the consent form were made in order to tailor it to the level of understanding of the youth being interviewed. For example, participants were provided with information about the study that was explained by the interviewer using pictures and written words. Picture cards such as a stop sign to indicate the need for a break and a question mark to indicate “I don’t understand” were presented. These picture cues were also made available throughout the interview. During both the assent process and the interview we provided as many options to support communication with the aim that participants would be able to use at least one of them to communicate with the interviewer. As suggested in previous research (Inglis & Cook, 2011), interviewers were also attentive to additional nonverbal cues, such as the participant getting up, putting their head on the table or moving toward the door. This was done to ensure continued assent and engagement in the interview, assess the need for breaks and decide when to end the interview.

Following consent/assent, the interview started with an activity where participants were asked to order five main themes (printed on cards with pictures and words) from least to most stressful. The ordered theme cards were left visible on the table and were subsequently discussed in sequence. This activity gave participants agency to structure the interview, making the interview more predictable for them. The *Leisure* and *Autism Experience* themes were explored independently from the ordering activity.

**Figure 1.**
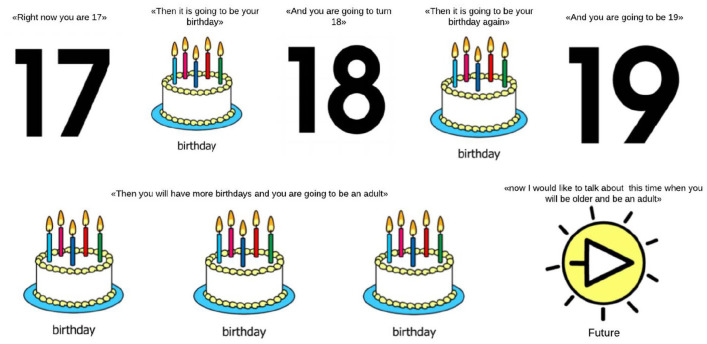
Future theme baseline presentation. Cards were placed one by one in front of the participant, while reading out the corresponding verbal prompt.

Each theme was first explored using standardized baseline questions suitable for all participants. These baseline questions were developed following universal design principles (Story, 1998). Universal design is a way to organize environments, products or services to allow all people to access and use them, without adaptations, and regardless of their sex, age, status, or disability (Goldsmith, 2012). Each baseline question was then explored using prompts and follow-up questions customized for each participants’ communication preference. See, as an example, Figure 1, which Illustrates how the Future theme was presented to all participants.

### Data collection

The interviews were conducted by one of four interviewers (RT, VC, WM, and a trained research assistant) either at the family home (*n* = 8) or a clinical research setting (*n* = 24), depending on the participant’s preference. The duration of the interviews was 60 to 90 min, depending on participants’ verbal ability and level of engagement. Interviews were video-recorded and then transcribed verbatim by a professional transcriptionist, including nonverbal content (e.g. affect, body movements, facial expression, voice tone, etc.). To ensure transcripts accurately reflected the content of the interview, the first few transcripts were reviewed by the team (including interviewers) and cross-validated with the video recordings as well as with the subjective experience of the interviewer. Only minor adjustments were made and the team felt confident that the transcripts accurately represented the content of the interviews.

## Data analysis

Following data collection, all interviewers discussed barriers and facilitators that occurred in their interviews with the rest of the research team (P.M., M.E., I.S., L.Z., D.N.). Further revision of recorded interviews was used during our discussion process and led to a list of salient factors that promoted or inhibited the implementation and engagement with the interview.

In addition, we developed codes to assess the suitability of the *Autism Voices* interview to capture first person perspectives of adolescents across the autism spectrum. Codes were developed to capture verbal and nonverbal communicative acts that also included strategies that were non-conventional or difficult to interpret. We also developed codes to identify mitigation strategies used by the interviewers in response to communication that was non-conventional/difficult to interpret. Such mitigation strategies can be effective in ensuring the accuracy of a response (Rodgers, 1999) or to facilitate the communication between interviewer and participant (see Table 3). We aimed to document how the participating teenagers and interviewers communicated with one another and which strategies were effective and efficient in enhancing understanding. Two coders initially reviewed a first transcript for any form of non-conventional communication observed in the session. The coders independently created an initial list of communicative acts, which were then compared and combined after reaching a consensus. Two additional transcripts were independently reviewed by the same coders and new communication codes agreed upon were added to the previous list. Review of the third transcript did not lead to the addition of new codes; hence, saturation was deemed to have been achieved. The codes were then reviewed by all authors to remove redundancy and increase the clarity of their definitions. Once finalized, the coding scheme was used by a coder blind to the study’s objectives. See Table 3 for a complete list of non-conventional communicative acts used by participants and mitigation strategies to address challenges to communication during the interview. A separate coding scheme will be developed to capture the content arising from participant’s responses and will be presented in a subsequent paper.

**Table 3.** Coding scheme including definition of (A) communicative acts used by participating adolescents and (B) mitigation strategies used by interviewers to address challenges to communication and facilitate capture of first-person perspectives.

A. Communicative acts	Definition
1. Absence of answer	
a. Provides no answer	No response to question
b. Refuses to answer	Explicit refusal to answer a question
2. Standing up/pacing	Moves away from the interviewer/table
3. Fixed pattern of response	Choosing the same category for all responses or always providing a positive or a negative response
4. Inconsistencies	Pair of codes: Stating something then its opposite later in the interview
5. Echolalia	
a. Reading/Written echolalia	Reading/ Writing words from cards provided as support for questions
b. Immediate echolalia	Repeating a portion of examiner’s questions
c. Delayed echolalia	Repeats a pre-learned phrase
6. Emphatic answer	No hesitation and quick very precise answer. Change in the emotional valence. Eagerness when providing an answer.
B. Mitigation strategies	Definition
1. Enforcing limits	Interviewer enforces boundaries (time, subject, etc.)
2. Acknowledging what participant is doing/saying	Rephrasing or repeating the answer or providing positive reinforcement following an answer
3. Asking the next question	Moving forward with the interview
4. Providing response choice to avoid echolalia or pattern of response	Giving options to force participant to respond in a non-echolalic or stereotyped way
5. Repeating/rephrasing question	Asking the question the same way or using synonyms
6. Prompting	
a. Expanding on the question	Providing help or clues to encourage participant to respond
b. Confirming understanding	Ensuring that participant understood the question
7. Offering a different output	Providing participant with an alternative output to respond

### Community involvement

Community involvement and co-production of knowledge with the autism community and their families are central to our research. As detailed in the previous section, our methods are informed by the lived experiences of parents caring for an autistic child. Further, during our piloting of these methods with a minimally-verbal participant, we considered the participant’s attitude, engagement level and non-verbal communication to assess the acceptability of our initial protocol. In addition, at the end of each interview we conducted, autistic participants were directly asked their thoughts on the interview process and what they would change. For minimally verbal participants, we paid particular attention to their body language and other non-verbal communication (e.g. pointing to cards, writing), to better gauge what sections of the interview they most enjoyed, which informed the sections we focused on. Feedback from autistic participants was embedded within protocol development and subsequent improvements, their feedback also guided the recommendations we are making in the “Discussion” section. As such, we believe that Autism Voices is a major contribution to the field of engagement because the study directly addresses inherent barriers in communication and contribution from autistic youth.

## Results

### Barriers and facilitators of the interview protocol

#### Question formulation

Overall, the questions selected and the approach taken were useful in generating responses from all participants. However, two forms of questions were particularly challenging; these were “wh” questions (who, what, where, when, why) and those that included a negative phrasing, such as “don’t like” and “can’t do.” Examples 1 and 2 are included in the following. The mitigation strategy most used by interviewers in response to this barrier was to repeat or rephrase the questions to make them more intelligible. See example 3 in the following.

Example 1. Interviewer: Where do you go to school?

Participant: Monday.

Example 2. Interviewer: Is there anything you don’t like at school?

Participant: I like it, school. Teacher.

Interviewer: OK. You like the teacher or you don’t like the teacher? Which one, choose?

Participant: I like it, teacher.

Example 3. Interviewer: How do you get to the city? What type of transportation?

Participant: (looks at interviewer contemplatively)

Interviewer: What type of transportation do you use?

Participant: (shakes head no)

Interviewer: OK so do you bike to Montreal?

Participant: No.

Interviewer: Do you take the car?

Participant: Yeah.

#### Use of pictures to ask the questions

Pictures were useful not only as one of the outputs offered (see Outputs offered, in the following), but also as a way for the interviewer to communicate with youth and structure the interview. While some participants with lower NVIQ scores and/or minimal verbal language likely would not have been able to participate without the use of pictures when asking questions, verbal participants with normal NVIQ scores often appeared to benefit from the pictures as well. For example, the ordering activity allowed the interviewer to lay the themes that were to be discussed on the table in front of the participant and to visually indicate when questions about a specific theme were done by turning the respective card over.

Pictures also helped in keeping the conversation on topic, as the interviewer would announce: “Now I would like to talk with you about X or Y.” They would then leave the theme card in front of the participant during the discussion which served as a reminder to remain on topic. For instance, one of our youth participants with limited verbal communication quickly returned his focus to the topic of discussion when the interviewer pointed to the picture. As previously done by others (Harrington et al., 2014; Nind, 2008; Stalker, 1998), offering picture examples was also used to elicit answers and facilitate communication with some verbal participants who had more difficulties generating original answers (e.g. providing different examples of potential job fields). Indeed, sometimes, participants did not yet know what specific job they wanted, but these categories helped them narrow down a field or exclude some fields they were not interested in.

#### Outputs offered

As mentioned above, multiple and varied response modalities were used by the teenagers to communicate (typing, writing, choosing images, pointing, verbally answering, spelling to communicate, and texting emojis). Interestingly, writing was used by two minimally verbal participants (one with low NVIQ and one with normal/high NVIQ). Both were interested in the text written below the pictures presented, and parents confirmed they enjoyed writing words they see. Offering varied output modalities allowed participants of all language levels to engage in the interview; such adaptations to a classic verbal interview were not only helpful, but necessary for some youth.

#### Presence of parents in the room

For four minimally verbal participants and for six of the younger participants (aged 11 to 13 years), the presence of one or both parents seemed necessary or preferred to complete the interview. Reasons for parents’ presence varied. One participant required assistance to use his communication device, another participant refused to sit down and participate when his father left the room, and others explicitly asked for their parents. During the interview, some parents noted that they were pleasantly surprised by their child’s ability to understand the questions and/or by the responses they provided. In some cases, parents also were able to confirm or clarify some of the answers provided by their child, which was useful to the interviewer. Conversely, several older participants who were verbal stated they did not want their parents present and felt more at ease talking without them. Hence, no “one size fits all” guideline can be provided regarding the inclusion of parents in the interview. The pros and cons of having parents in the room should be carefully considered on a case-by-case basis.

#### Flexibility of the interviewer

Interviewers differed slightly in how they followed the semi-structured interview protocol and how they used mitigation strategies. For example, interviewers that were more flexible in their approach would provide more prompts instead of asking a next question, which led to richer information and more informal conversation-like interviews. Furthermore, interviewers who tended to follow the interview questions more strictly would ask questions that were previously answered from another theme. This oversight was noted by some participants who responded with statements such as “*I told you already*.” Overall, more flexibility during the interview lead to notably better rapport with the participant, which in turn, seemed to lead to an overall more positive experience for the participant. This was observed as less agitation toward the end of the interview, less eagerness to finish the interview, and a better subjective experience reported by participants.

### Alternative communication acts and mitigation strategies used

Relative to other participants, interviews with minimally verbal participants and participants with lower NVIQ scores were characterized by more frequent use of alternative communication acts (e.g. no response, rigid or scripted response, echolalia, etc.). However, a finer analysis of these communication acts suggested that they should not be considered as barriers to standard communication, as they also represented a way to communicate and engage with the interviewer. As for mitigation strategies, all strategies listed in Table 3 were used frequently and spontaneously by interviewers. Mitigation strategies were used most with minimally verbal participants as they used alternative communication acts more frequently. The success of mitigation strategies to obtaining a conventional answer to a question was mixed. Examples of successful strategies are described in the following.

#### Learned behaviors and social desirability

One of the least frequently observed alternative communication acts was refusal to answer questions. When participants did refuse to answer, it was often tied with questions about negative emotions, for example, “what makes you sad,” “what worries you,” “what don’t you like?,” and so on. A non-answer did not necessarily translate to a direct refusal to answer questions; rather, some participants redirected answers for things that they liked. In line with this observation, participants often put “everything” in the “like” category or the “not worried” piles of cards.

Another possible social desirability effect is the fixed pattern of response that was observed mostly in minimally verbal participants. Participants tended to use “yes” as a patterned response to close-ended questions or continued to add more items to the “I like” pile when prompts such as “what else do you like?” were used. See example 4 in the following. The mitigation strategy most often used in response to this pattern of response was *providing an answer choice to avoid patterns of response*, as illustrated in example 5 in the following.

Example 4. Interviewer: OK. So that’s family?

Participant: Yes.

Interviewer: Do you have any other family?

Participant: Yes.

Interviewer: Who, who else? Do you want to draw them?

Participant: Yes.

Interviewer: OK. Can you draw them?

Participant: Yes. (picks up pen, but does not draw).

Interviewer: Who else?

Participant: Yes.

Example 5. Interviewer: Who else [is in your family]?

Participant: Yes.

Interviewer: Do you have a brother?

Participant: Yes.

Interviewer: OK. What’s your brother’s name?

Participant: (says brother’s name).

#### Communication goes beyond conventional verbal responses

The most common form of alternative communication used was echolalia (i.e. repeating words previously heard, either immediately or after a delay). Close examination of the transcripts highlighted that a participant’s echolalic responses were a means to actively communicate different forms of responses. For example, use of delayed (example 6) or immediate (example 7) echolalia was a reflection of the youth’s interests and was used as a way to engage in the interview:

Example 6. Interviewer: OK. Who else is in your family? Who else?

Participant: Family Guy.

Interviewer: So we have (says sister’s name). OK. And then we have dad. Yes? Dad was right here? So we have sister, and dad. And then who else?

Participant: Baby Einstein.

Example 7. Interviewer: OK, [you like] iPad/tablet. [what about] Making movies–

Participant: Making movies.

Interviewer: You like making movies?

Participant: Yes

Interviewer: You like movies?

Participant: Movies, your favourite.

Interviewer: What’s your favourite movie?

Participant: Favourite movie.

Interviewer: What do you like?

Participant: Movie you like? (leans forward, touches interviewer’s hand)

Interviewer: I like Harry Potter.

Participant: Yes. Harry Potter. Cinema, going to see a movie.

Participants’ emphatic responses offered an effective means for the youth to communicate about a particular theme of interest. For instance, in the example below, the minimally verbal participant had generated very few original answers. However, when asked about what he wanted in terms of a job in the future, he generated a clear and detailed answer:

Example 8. Interviewer: Great. And what about jobs? What does (participant) want to do when he is an adult?

Participant: Yes.

Interviewer: What do you want to do [for a job as an adult]?

Participant: Building a cinema.

Interviewer: You want to build a cinema.

Participant: Yes.

Interviewer: Oh cool. What would you put in the cinema? How many theatres would you have?

Participant: Nine screens, featuring ultra AVX.

Interviewer: Whoa, so it would be like 9 really big screens you would build. You would build 9 big screens in your cinema?

Participant: Yes and 10 ultra AVX and 11 IMAX.

## Discussion

Based on previous literature and on our synthesis of methods used to capture first person perspectives of youth with disabilities (Tesfaye et al., 2019), we developed a strength-oriented methodology; *Autism Voices* to capture the lived experience of autistic adolescents. Our protocol included a pre-interview parent survey and a semi-structured interview, alongside a coding scheme to systematically interpret communication between the participant and interviewer and maximize capture of first-person perspectives. Universal design principles guided the development of the method so that it could be as inclusive as possible, enabling autistic adolescents regardless of their language abilities or intelligence level to participate. The parental pre-interview survey allowed us to collect relevant information about the youth and to document their preferred ways of communicating, which was used to tailor interviews. During the interview, six themes were explored: school, family, friends, future, leisure, and experience with autism. Questions were presented using pictorial supports for everyone and a variety of outputs were offered for the youth to respond (writing, texting, drawing, typing, pointing, talking, etc.). Using this novel methodology, we demonstrated that eliciting the lived experience of autistic youth across the spectrum is possible. However, as discussed in the following, it requires time, active preparation, and flexibility.

Substantial effort went into the preparation and tailoring of each interview, and key factors were needed to successfully conduct participatory research with youth with complex communication needs (Inglis & Cook, 2011; Stalker, 1998). First, as was the case in previous studies, the parental pre-interview phone survey helped determine how best to facilitate each youth’s participation (Carroll & Sixsmith, 2016). For example, the best setting to allow for a comfortable and successful interview was discussed with the parent. In our previous synthesis, among the preferable settings identified, few were in clinical or laboratory settings (Tesfaye et al., 2019). Hence, we anticipated that home settings or other familiar settings to youth (e.g. school) would be preferred. However, many parents suggested that the interview might be less successful at home given contextual drawbacks (e.g. distractions, no quiet room available, etc.). Hence, most of the interviews were conducted in a clinic setting unless requested at home. Second, as anticipated based on the results of our synthesis, parents’ presence in the interview room ended up being necessary and particularly helpful for minimally verbal participants and for some younger participants to facilitate communication, but was not always necessary nor helpful (Rodgers, 1999), especially for more verbal participants.

As highlighted in previous research, parents were sometimes needed to facilitate communication or used as a proxy for their minimally verbal children (Hemsley et al., 2013; Kirk, 2010; Lindsay & McPherson, 2012). However, when interviewing minimally verbal autistic youth with a proxy (parent, teacher, siblings, etc.), the possibility that the proxy may interject their own perspectives or that their presence may alter participant’s responses needs to be considered (Preece & Jordan, 2010). Conversely, verbal and/or older participants tended to be more comfortable disclosing private information without their parent present. The aforementioned arguments for and against a parent’s presence in the interview room leads us to favor the conditions in which the interview will be richer and most comfortable for the youth and this cannot always be determined in advance (Rodgers, 1999). The third aspect of the interview that was tailored was the response outputs offered to the youth. We found it useful to have a few targeted resources that were identified as potential facilitation techniques in our synthesis (Tesfaye et al., 2019). For example, a few minimally verbal participants started to write words when provided with a pencil and paper, although writing was not identified previously as a preferred method of communication by a parent. This interest for writing is consistent with previous studies demonstrating autistic children develop an intense interest for writing early in development (Jacques et al., 2018), which could be a promising avenue to communicate with minimally verbal autistic youth (Ostrolenk et al., 2017). Furthermore, while in our synthesis other facilitators such as emails or instant messaging were suggested (e.g. Bottema-Beutel et al., 2016), to our knowledge this is the first study to successfully utilize texting and emojis in a personal interview. Given the ubiquitous nature of smartphones and emoji use with teenagers, this may serve as a useful communication option in interviews.

### Semi-structured interview protocol

Our results highlight that question formulation is key to ensure youth understand what the interviewer is asking, and to facilitate the interviewers’ understanding of their responses. Open-ended question, especially the “wh” questions are particularly challenging for youth with language difficulties (Booth & Booth, 1996). When re-asking a question, reformulating using synonyms, adding the use of labeled pictures, and/or writing parts of the question often helped participants to better grasp what the interviewer was interested in knowing. This resulted in guiding youth to provide answers that were in line with the questions asked. Previous analyses show that elicitation and facilitation techniques allow access to information that would otherwise be missed (Teachman & Gibson, 2013) and our study confirms this. Another key aspect was interview flexibility. Flexibility included skipping a question and coming back to it later, removing questions that were previously covered to some extent, allowing a participant to stand and/or pace when answering, adding follow-up questions when a participant’s answer warranted more exploration, and offering different outputs. Such flexible administration allowed for the interviews to be conversation-like instead of being assessment-oriented. Although, we did not specifically code for interviewer–interviewee interactions, this could be an interesting future direction to further inform how to promote reciprocal interactions with autistic youth (Fusaroli et al., 2019). Indeed, there could be many barriers to effective communication between autistics and non-autistics and they are currently poorly understood and mostly studied from the autistic deficit perspective instead of from an interactional/reciprocal perspective (Edey et al., 2016).

We also observed that spontaneous mitigation strategies could be used by interviewers to broaden perspectives, which could inform future approaches to conducting interviews with this population. Finally, the use of a universal baseline presentation for all participants not only served to ensure a baseline set of questions were common to all participants but also helped to structure the interview.

This study has highlighted the often-underestimated ability and communication of autistic teenagers—especially minimally verbal autistic teenagers and those with lower NVIQ scores. Our communication codes and processes allowed for an in-depth analysis of what otherwise could have been considered unsuccessful interviews. Rigidity in research and under-estimating participants’ potential to engage in research can limit us to only conventional question–response interactions (Rodgers, 1999; Stalker & Connors, 2003). Our fine-grained analysis showed that atypical communication *is* communication and it often has an important message behind it. This invites researchers to be open to alternative ways of information sharing, opening spaces for participants—irrespective of autism and IQ expression—to participate in first-person experience-based research. As illustrated in this study, all of the participants were engaged in the interview and actively interacted with the interviewer, leading us to conclude that the participants were always trying to communicate something. Even the few refusals to answer questions or silences provided useful information about topics participants did not want to discuss (Booth & Booth, 1996). Furthermore, echolalia, which was one of the most frequent communication acts observed in our study, was shown to have a communicative and interactive purpose (Sterponi & Shankey, 2014). Here, immediate echolalia showed that the participant was listening and processing what the interviewer was saying, while delayed echolalia at times communicated salient areas of interest. Topics of interests were also often communicated through emphatic answers and represent a promising window to enable communication with these youth (Jordan & Caldwell-Harris, 2012). Assuming ability is a prerequisite for participatory research with youth with complex needs in general (Inglis & Cook, 2011; Rodgers, 1999) and is of paramount importance to promote participatory research in autism.

If our research protocol had not intentionally included minimally verbal and intellectually disabled autistic participants, many of our participants would have been excluded based on their communication limitations (Stafford, 2017). Indeed, even parents of some of the participants reported prior failed attempts to have their child participate in various research and several initially were reluctant to participate in this study as result of these negative experiences. As a result, they were pleased that their child had meaningfully participated and were sometimes surprised by their responses. It is often the case when conducting research with youth that are considered disabled that they are perceived to exceed expectations (Rodgers, 1999). In our study, for example, several parents stated they had never considered that their child thought about where they would live in the future and were pleased to learn about this. Studies capturing parent’s perspectives show they are highly concerned about their child’s future and, despite wanting them to live independently, report their children do not fully grasp what that entails (Cribb et al., 2019; Poon et al., 2013; Sosnowy et al., 2018). In contrast, when autistic youth were interviewed themselves, they reported being aware of at least some of the challenges they would face to reach independence and were confident in their ability to reach this goal if given support (Cribb et al., 2019; Sosnowy et al., 2018). Hence, regardless of families’ or researchers’ perceptions of youth ability, we caution that ability should always be assumed as participants often exceed expectations from others (Inglis & Cook, 2011). This approach will allow researchers to access youth’s maximal potential.

Supplemental efforts are needed to fully grasp non-conventional communication and go beyond potential learned behavior or social desirability in answers. For example, in an earlier consultation (Tesfaye et al., 2019), parents had advised us to avoid questions about emotions because their child would not understand them. However, in the present study, participating adolescents were mostly capable of answering emotion-based questions and had things to say about what made them feel sad, angry, stressed, or happy. As researchers and the broader community, it should not be assumed that people across the autism spectrum do not have an opinion about important matters in their lives. Rather, we need to find better ways to respectfully inquire and engage (Fletcher-Watson et al., 2018).

### Limits and potential improvements to Autism Voices

The study highlighted several potential opportunities to improve the *Autism Voices* protocol. For instance, it became quickly evident that “wh” questions and use of negation makes questions harder to interpret. Pairing any question with picture cards and written words enhances understandability. Regarding mitigation strategies, prompting for a more detailed answer by using probes such as “is there anything else you like at school?” or “what else do you like?” was not as effective as expected. This method sometimes led to the participant providing as many answers as they were prompted, thus leading to what appeared as less authentic answers. It is possible that some minimally verbal participants may have learned that answering a consistent response (e.g. yes) to questions, most often leads to a better outcome or is what is expected from others. Therefore, as previously observed in research with youth with disabilities (Rodgers, 1999; Stalker, 1998), some participants were prone to providing such patterned responses. Furthermore, with minimally verbal participants, it was often necessary for the interviewer to provide picture cards with specific examples of answers, which could bias some of the participants’ answers. Where the options are easy to list, providing response options was, however, effective in avoiding patterned responses. Similarly, asking for a fixed number of items, for example, “tell me three things you like about school,” seemed to provide scaffolding for the participants and facilitate a response. As for verbal participants, providing picture cards with examples of categories or fields probing them to reflect on questions they may not have thought about previously was also helpful to narrow down fields in which they would like or not like to work. Using such prompts, only when the participant does not generate an original answer, can be useful to explore the question further.

Another interesting pattern worth further exploration in future studies is the refusal to answer questions related to “negative” emotions. It is unclear whether this is because the participants felt uneasy talking about these topics or rather that they learned to avoid them based on expectations from others, but this unease or refusal to discuss “negative” emotions was also observed by others (Booth & Booth, 1996; Richards & Crane, 2020). While it can be distressing and challenging for anyone to talk about unpleasant emotions, if there also is a social desirability factor in play here and it turns out to be pervasive in the socialization of autistic youth, then authentic answers related to negative topics may be particularly challenging to obtain. This warrants further investigation. However, some promising innovative methods such as offering autistic youth the possibility to write their negative feelings and experiences and then place them in an envelope, instead of talking about them were recently proposed (Richards & Crane, 2020).

Finally, given the amount of content we covered, conducting the interview over more than one session and potentially in more than one setting would be beneficial. This would allow us to account for variability in their responses due to location, events, mood or unfamiliarity with the interviewer. Getting to know the participant before starting to collect data is recommended (Stalker, 1998) and could have enhanced the quality of the *Autism Voices* interviews. Ideally, more precise methods to further tailor the interviews for each participant would offer maximal opportunities for self-expression (Abbott, 2013; Stafford, 2017; Stalker & Connors, 2003). For example, an introductory interview in the family home where the participant, family and interviewer get acquainted in the familiar setting of the home could be followed by a subsequent interview in a lab/clinical setting or during a walk in the park.

## Conclusion

*Autism Voices* provides a step toward creating inclusive methodologies within autism and other disability research. No specific training is necessary to use our method, but familiarity and experience with autistic people as well as some clinical experience or experience conducting semi-structured interviews are warranted. By sharing our methodology and approach, we invite the research community to build on the principles of *Autism Voices* to design inclusive methods that can be used with autistic children, adolescents, adults or even for other individual’s with complex communication needs. This inclusive methodology can have a broader impact and be beneficial within many settings beyond research (e.g. education, healthcare, community services, etc.) A key take-away of *Autism Voices* is youth with various abilities are capable of voicing their perspectives if the community meets them where they are at. This methodology and approach to engagement will ultimately lead to the empowerment of the autistic community and will promote their self-determination by including them as active stakeholders in research that affects them.

## Supplemental Material

sj-docx-1-aut-10.1177_13623613211042128 – Supplemental material for Autism Voices: A novel method to access first-person perspective of autistic youth Supplemental material, sj-docx-1-aut-10.1177_13623613211042128 for Autism Voices: A novel method to access first-person perspective of autistic youth by Valérie Courchesne, Rackeb Tesfaye, Pat Mirenda, David Nicholas, Wendy Mitchell, Ilina Singh, Lonnie Zwaigenbaum and Mayada Elsabbagh in Autism

## References

[bibr1-13623613211042128] Abbott D. (2013). Who says what, where, why and how? Doing real-world research with disabled children, young people and family members. In Curran T. Runswick-Cole K. (Eds.), Disabled children’s childhood studies (pp. 39–56). Springer.

[bibr2-13623613211042128] American Psychiatric Association. (2000). Diagnostic and statistical manual of mental disorders (4th ed., text rev.). Washington, DC.

[bibr3-13623613211042128] American Psychiatric Association. (2013). Diagnostic and statistical manual of mental disorders (5th ed.). American Psychiatric Publishing.

[bibr4-13623613211042128] Andersen C. S. Dolva A.-S. (2015). Children’s perspective on their right to participate in decision-making according to the United Nations Convention on the Rights of the Child article 12. Physical & Occupational Therapy in Pediatrics, 35(3), 218–230. 10.3109/01942638.2014.918075 24865121

[bibr5-13623613211042128] Bailey S. Boddy K. Briscoe S. Morris C. (2015). Involving disabled children and young people as partners in research: A systematic review. Child: Care, Health and Development, 41(4), 505–514. 25323964 10.1111/cch.12197

[bibr6-13623613211042128] Bieleninik Ł. Posserud M.-B. Geretsegger M. Thompson G. Elefant C. Gold C. (2017). Tracing the temporal stability of autism spectrum diagnosis and severity as measured by the Autism Diagnostic Observation Schedule: A systematic review and meta-analysis. PLOS ONE, 12(9), Article e0183160. 10.1371/journal.pone.0183160 PMC5608197 28934215

[bibr7-13623613211042128] Booth T. Booth W. (1996). Sounds of silence: Narrative research with inarticulate subjects. Disability & Society, 11(1), 55–70.

[bibr8-13623613211042128] Bottema-Beutel K. Mullins T. S. Harvey M. N. Gustafson J. R. Carter E. W. (2016). Avoiding the “brick wall of awkward”: Perspectives of youth with autism spectrum disorder on social-focused intervention practices. Autism, 20(2), 196–206. 25882390 10.1177/1362361315574888

[bibr9-13623613211042128] Bronfenbrenner U. (1989). Ecological systems theory. In Vasta R. (Ed.), Annals of child development: Vol. 6. Six theories of child development: Revised reformulations and current issues, (pp. 187–250). JAI Press.

[bibr10-13623613211042128] Carrington S. J. Uljarević M. Roberts A. White L. J. Morgan L. Wimpory D. . . . Leekam S. R. (2016). Knowledge acquisition and research evidence in autism: Researcher and practitioner perspectives and engagement. Research in Developmental Disabilities, 51, 126–134. 26826464 10.1016/j.ridd.2016.01.011

[bibr11-13623613211042128] Carroll C. Sixsmith J. (2016). Exploring the facilitation of young children with disabilities in research about their early intervention service. Child Language Teaching and Therapy, 32(3), 313–325.

[bibr12-13623613211042128] Charlton J. I. (2000). Nothing about us without us: Disability oppression and empowerment. University of California Press.

[bibr13-13623613211042128] Chown N. Robinson J. Beardon L. Downing J. Hughes L. Leatherland J. . . . MacGregor D. (2017). Improving research about us, with us: A draft framework for inclusive autism research. Disability & Society, 32(5), 720–734.

[bibr14-13623613211042128] Cribb S. Kenny L. Pellicano E. (2019). “I definitely feel more in control of my life”: The perspectives of young autistic people and their parents on emerging adulthood. Autism, 23(7), 1765–1781. 30818981 10.1177/1362361319830029

[bibr15-13623613211042128] Deci E. L. Ryan R. M. (2004). Handbook of self-determination research. University Rochester Press.

[bibr16-13623613211042128] DePape A.-M. Lindsay S. (2016). Lived experiences from the perspective of individuals with autism spectrum disorder: A qualitative meta-synthesis. Focus on Autism and Other Developmental Disabilities, 31(1), 60–71.

[bibr17-13623613211042128] Edey R. Cook J. Brewer R. Johnson M. H. Bird G. Press C. (2016). Interaction takes two: Typical adults exhibit mind-blindness towards those with autism spectrum disorder. Journal of Abnormal Psychology, 125(7), 879–885. 27583766 10.1037/abn0000199

[bibr18-13623613211042128] Fletcher-Watson S. Adams J. Brook K. Charman T. Crane L. Cusack J. . . . Pellicano E. (2018). Making the future together: Shaping autism research through meaningful participation. Autism, 23(4), 943–953. 30095277 10.1177/1362361318786721 PMC6512245

[bibr19-13623613211042128] Fusaroli R. Weed E. Fein D. Naigles L. (2019). Hearing me hearing you: Reciprocal effects between child and parent language in autism and typical development. Cognition, 183, 1–18. 30396129 10.1016/j.cognition.2018.10.022 PMC6322977

[bibr20-13623613211042128] Gal T. (2017). An ecological model of child and youth participation. Children and Youth Services Review, 79, 57–64.

[bibr21-13623613211042128] Goldsmith S. (2012). Designing for the disabled: The new paradigm. Routledge.

[bibr22-13623613211042128] Graetz J. E. (2010). Autism grows up: Opportunities for adults with autism. Disability & Society, 25(1), 33–47.

[bibr23-13623613211042128] Grinker R. R. Chambers N. Njongwe N. Lagman A. E. Guthrie W. Stronach S. . . . Chhagan M. (2012). “Communities” in community engagement: Lessons learned from autism research in South Korea and South Africa. Autism Research, 5(3), 201–210. 22566396 10.1002/aur.1229 PMC3552431

[bibr24-13623613211042128] Harrington C. Foster M. Rodger S. Ashburner J. (2014). Engaging young people with autism spectrum disorder in research interviews. British Journal of Learning Disabilities, 42(2), 153–161.

[bibr25-13623613211042128] Hemsley B. Kuek M. Bastock K. Scarinci N. Davidson B. (2013). Parents and children with cerebral palsy discuss communication needs in hospital. Developmental Neurorehabilitation, 16(6), 363–374. 24228709 10.3109/17518423.2012.758187

[bibr26-13623613211042128] Inglis P. Cook T. (2011). Ten top tips for effectively involving people with a learning disability in research. Journal of Learning Disabilities and Offending Behaviour, 2, 98–104.

[bibr27-13623613211042128] Jack A. Pelphrey K. A. (2017). Annual Research Review: Understudied populations within the autism spectrum–current trends and future directions in neuroimaging research. Journal of Child Psychology and Psychiatry, 58(4), 411–435. 28102566 10.1111/jcpp.12687 PMC5367938

[bibr28-13623613211042128] Jacques C. Courchesne V. Meilleur A.-A. S. Mineau S. Ferguson S. Cousineau D. . . . Mottron L. (2018). What interests young autistic children? An exploratory study of object exploration and repetitive behavior. PLOS ONE, 13(12), Article e0209251. 10.1371/journal.pone.0209251 PMC6312372 30596684

[bibr29-13623613211042128] Jordan C. J. Caldwell-Harris C. L. (2012). Understanding differences in neurotypical and autism spectrum special interests through internet forums. Intellectual and Developmental Disabilities, 50(5), 391–402. 23025641 10.1352/1934-9556-50.5.391

[bibr30-13623613211042128] Kasari C. Brady N. Lord C. Tager-Flusberg H. (2013). Assessing the minimally verbal school-aged child with autism spectrum disorder. Autism Research, 6(6), 479–493. 10.1002/aur.1334 24353165 PMC4139180

[bibr31-13623613211042128] Kirk S. (2010). How children and young people construct and negotiate living with medical technology. Social Science & Medicine, 71(10), 1796–1803. 20933314 10.1016/j.socscimed.2010.07.044

[bibr32-13623613211042128] Lebenhagen C. (2019). Including speaking and nonspeaking autistic voice in research. Autism in Adulthood, 2(2), 128–131. http://doi.org/10.1089/aut.2019.0002 10.1089/aut.2019.0002 PMC8992839 36601567

[bibr33-13623613211042128] Lindsay S. McPherson A. C. (2012). Experiences of social exclusion and bullying at school among children and youth with cerebral palsy. Disability and Rehabilitation, 34(2), 101–109. 21870932 10.3109/09638288.2011.587086

[bibr34-13623613211042128] Lord C. Risi S. Lambrecht L. Cook E. H. Jr. Leventhal B. L. DiLavore P. C. . . . Rutter M. (2000). The Autism Diagnostic Observation Schedule—Generic: A standard measure of social and communication deficits associated with the spectrum of autism. Journal of Autism and Developmental Disorders, 30(3), 205–223. 11055457

[bibr35-13623613211042128] Lord C. Rutter M. Le Couteur A. (1994). Autism Diagnostic Interview-Revised: A revised version of a diagnostic interview for caregivers of individuals with possible pervasive developmental disorders. Journal of Autism and Developmental Disorders, 24(5), 659–685. 7814313 10.1007/BF02172145

[bibr36-13623613211042128] Minkes J. Townsley R. Weston C. Williams C. Tyrell J. (1995). Having a voice: Involving people with learning difficulties in research. British Journal of Learning Disabilities, 23(3), 94–97.

[bibr37-13623613211042128] Nicholas D. B. Orjasaeter J. D. Zwaigenbaum L. (2019). Considering methodological accommodation to the diversity of ASD: A realist synthesis review of data collection methods for examining first-person experiences. Review Journal of Autism and Developmental Disorders, 6(2), 216–232.

[bibr38-13623613211042128] Nind M. (2008, November). Conducting qualitative research with people with learning, communication and other disabilities: Methodological challenges. This paper is a review commissionned by the National Center for Research Methods which is part of the economic and social research concil.

[bibr39-13623613211042128] Ostrolenk A. Forgeot d’Arc B. Jelenic P. Samson F. Mottron L. (2017). Hyperlexia: Systematic review, neurocognitive modelling, and outcome. Neuroscience & Biobehavioral Reviews, 79, 134–149. 28478182 10.1016/j.neubiorev.2017.04.029

[bibr40-13623613211042128] Parr J. (2016). How can we learn more about the lives of adults on the autism spectrum from across the age range, and their relatives. In Wright Scott D. (Ed.), Autism spectrum disorder in mid and later life (pp. 288–296). Jessica Kingsley Publishers.

[bibr41-13623613211042128] Parsons S. Cobb S. (2013). Who chooses what I need? Child voice and user-involvement in the development of learning technologies for children with autism. EPSRC Observatory for Responsible Innovation in ICT.

[bibr42-13623613211042128] Perry-Hazan L. (2016). Children’s participation in national policymaking:“You’re so adorable, adorable, adorable! I’m speechless; So much fun!” Children and Youth Services Review, 67, 105–113.

[bibr43-13623613211042128] Poon K. K. Koh L. Magiati I. (2013). Parental perspectives on the importance and likelihood of adult outcomes for children with autism spectrum disorders, intellectual disabilities or multiple disabilities. Research in Autism Spectrum Disorders, 7(2), 382–390.

[bibr44-13623613211042128] Preece D. Jordan R. (2010). Obtaining the views of children and young people with autism spectrum disorders about their experience of daily life and social care support. British Journal of Learning Disabilities, 38(1), 10–20.

[bibr45-13623613211042128] Richards N. Crane L. (2020). The development and feasibility study of a multimodal “talking wall” to facilitate the voice of young people with autism and complex needs: A case study in a specialist residential school. Journal of Autism and Developmental Disorders, 50(12), 4267–4279. 32270384 10.1007/s10803-020-04476-6 PMC7677153

[bibr46-13623613211042128] Rodgers J. (1999). Trying to get it right: Undertaking research involving people with learning difficulties. Disability & Society, 14(4), 421–433.

[bibr47-13623613211042128] Russell G. Kapp S. K. Elliott D. Elphick C. Gwernan-Jones R. Owens C. (2019). Mapping the autistic advantage from the accounts of adults diagnosed with autism: A qualitative study. Autism in Adulthood, 1(2), 124–133. 31058260 10.1089/aut.2018.0035 PMC6493410

[bibr48-13623613211042128] Singh I. (2011). A disorder of anger and aggression: Children’s perspectives on attention deficit/hyperactivity disorder in the UK. Social Science & Medicine, 73(6), 889–896. 21684645 10.1016/j.socscimed.2011.03.049 PMC3176909

[bibr49-13623613211042128] Sosnowy C. Silverman C. Shattuck P. (2018). Parents’ and young adults’ perspectives on transition outcomes for young adults with autism. Autism, 22(1), 29–39. 29020791 10.1177/1362361317699585

[bibr50-13623613211042128] Stafford L. (2017). “What about my voice”: Emancipating the voices of children with disabilities through participant-centred methods. Children’s Geographies, 15(5), 600–613.

[bibr51-13623613211042128] Stalker K. (1998). Some ethical and methodological issues in research with people with learning difficulties. Disability & Society, 13(1), 5–19.

[bibr52-13623613211042128] Stalker K. Connors C. (2003). Communicating with disabled children. Adoption & Fostering, 27(1), 26–35.

[bibr53-13623613211042128] Sterponi L. Shankey J. (2014). Rethinking echolalia: Repetition as interactional resource in the communication of a child with autism. Journal of Child Language, 41(2), 275–304. 23469804 10.1017/S0305000912000682

[bibr54-13623613211042128] Story M. F. (1998). Maximizing usability: The principles of universal design. Assistive Technology, 10(1), 4–12. 10181150 10.1080/10400435.1998.10131955

[bibr55-13623613211042128] Teachman G. Gibson B. E. (2013). Children and youth with disabilities: Innovative methods for single qualitative interviews. Qualitative Health Research, 23(2), 264–274. 23208200 10.1177/1049732312468063

[bibr56-13623613211042128] Tesfaye R. Courchesne V. Yusuf A. Savion-Lemieux T. Singh I. Shikako-Thomas K. . . . Nicholas D. (2019). Assuming ability of youth with autism: Synthesis of methods capturing the first-person perspectives of children and youth with disabilities. Autism, 23(8), 1882–1896. 30915852 10.1177/1362361319831487 PMC6779014

[bibr57-13623613211042128] United Nations Children’s Fund. (1989). Convention on the rights of the child.

[bibr58-13623613211042128] World Health Organization. (2002). Towards a common language for functioning, disability and health, ICF: The international classification of functioning, disability and health.

