# Supplementary material for: Autism Voices: A novel method to access first-person perspective of autistic youth
Source: Autism. 2021 Sep 4;26(5):1123–36. doi: 10.1177/13623613211042128 (PMC9340132; doi:10.1177/13623613211042128)
Supplement: sj-docx-1-aut-10.1177_13623613211042128 – Supplemental material for Autism Voices: A novel method to access first-person perspective of autistic youth [file sj-docx-1-aut-10.1177_13623613211042128.docx]

ASD Voices Prep Visit

*Pathways III Page 1 of 2*

Individual ID

(Pathways Study Individual ID)

**Participatory Survey**

Preamble (on the phone): This Survey is meant to help us understand how your child likes to communicate best. As an expert on their capabilities, we need your insight in determining optimal strategies and approaches to gaining their perspectives and experience in a comfortable and authentic way.

Date of visit

**Section 1 Communication modality**

What is your child's preferred method of communication with you or others?

(*Probe: for instance, how do they respond to a question?)

Does your child require assistance or a device to

communicate? (e.g., alphabet/communication boards, speech devices, parent, speech therapist, specific

device)

If so, please specify

I'm going to go through a list of methods youth may use to communicate, please indicate if your child would be comfortable using:

Oral language (open ended conversation) Written word (i.e., writing, texting, typing) Drawing

Sorting images in order or in category piles

(e.g., we show them a picture of seasons and ask them to put them into an "I like" "I don't like"

or not sure section? Or favorite to least favorite) Pointing at images (or items)

Circling options

*Administrator note: select the methods already mentioned in question 1, do not repeat.

**Section 2 Interviewer**

In your opinion, who is your child most comfortable Researcher or clinician communicating with? Parent

Sibling or family member Educator


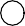

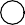

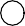

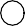

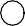


A friend

*Page 2 of 2*

**Section 3 Disclosure**

Have you disclosed your child's diagnosis to them? Yes No

Do they feel comfortable talking about their Yes

diagnosis? No


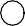

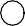

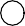

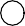


*In regards to the above questions, please ensure the parent/guardian that children's diagnoses nor the term ASD will be mentioned or discussed, unless the child brings it up themselves.

**Section 4 Interests**

List a few topics, activities (e.g., games), food, or interests your child enjoys. What do they like to talk about?

Are there certain topics {name} finds uncomfortable to talk about?

Who does [t1_arm_1][first_name] currently live with? (Pets?)

Who are people in [t1_arm_1][first_name] lifethat [she/he] is close to or would consider friends? (People that would be brought up in an interview).

Is there anyone in your child's life who we haven't Yes

discussed who they may like talking about or bring No up?


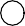

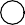


Please Specify

*Wrap up. Thank the parents for their time and ask if they have any questions for us at this time.
